# Integrated analysis of single-cell and bulk RNA-sequencing reveals the poor prognostic value of ABCA1 in gastric adenocarcinoma

**DOI:** 10.1007/s12672-023-00807-y

**Published:** 2023-10-24

**Authors:** Kaiyu Shen, Shuaiyi Ke, Binyu Chen, Wencang Gao

**Affiliations:** 1 The Second Clinical Medical College, Zhejiang Chinese Medical University, Hangzhou, 310053 China; 2 Department of Internal Medicine, Affiliated Xianju’s Hospital, XianJu People’s Hospital, Zhejiang Southeast Campus of Zhejiang Provincial People’s Hospital, Hangzhou Medical College, XianJu, 317399 China; 3 Department of Oncology, the Second Affiliated Hospital, Zhejiang Chinese Medical University, Hangzhou, 310005 China

**Keywords:** ABCA1, Gastric adenocarcinoma, Bioinformatics, Biomarker, Tumor-Associated macrophages

## Abstract

**Purpose:**

ATP-binding cassette A1 (ABCA1) is a potential prognostic marker for various tumor types. However, the biological effects and prognostic value of ABCA1 in gastric adenocarcinoma (GAC) remain unknown.

**Methods:**

GAC-associated single-cell RNA and bulk RNA-sequencing (bulk-seq) data were obtained from the Gene Expression Omnibus and The Cancer Genome Atlas databases, respectively. The differential expression of ABCA1 between GAC and normal gastric tissues was analyzed based on the bulk-seq data. Additionally, the relationship between ABCA1 expression and various clinicopathological features was explored. Furthermore, Kaplan–Meier survival and Cox regression analyses were performed to establish the prognostic value of ABCA1. The relationships between ABCA1 expression and anti-tumor drug sensitivity and immune checkpoints were also explored. Finally, the biological functions of ABCA1 were evaluated at the single-cell level, and in vitro studies were performed to assess the effects of ABCA1 on GAC cell proliferation and invasion.

**Results:**

ABCA1 expression is significantly elevated in GAC samples compared with that in normal gastric tissues. Clinical features and survival analysis revealed that high ABCA1 expression is associated with poor clinical phenotypes and prognosis, whereas Cox analysis identified ABCA1 as an independent risk factor for patients with GAC. Furthermore, high ABCA1 expression suppresses sensitivity to various chemotherapeutic drugs, including cisplatin and mitomycin, while upregulating immune checkpoints. ABCA1-overexpressing macrophages are associated with adverse clinical phenotypes in GAC and express unique ligand–receptor pairs that drive GAC progression. In vitro, ABCA1-knockdown GAC cells exhibit significantly inhibited proliferative and invasive properties.

**Conclusion:**

High ABCA1 expression promotes an adverse immune microenvironment and low survival rates in patients with GAC. Furthermore, ABCA1 and ABCA1-producing macrophages may serve as novel molecular targets in GAC treatment.

**Supplementary Information:**

The online version contains supplementary material available at 10.1007/s12672-023-00807-y.

## Introduction

Owing to its atypical symptoms and insidious onset, gastric adenocarcinoma (GAC) is a global public health issue that endangers human health with high morbidity and mortality rates [1, 2]. However, early diagnosis and treatment can improve the five-year survival rate to 90–97% [3]. Compared to conventional gastroscopy, biomarker testing is simpler and less costly to implement in early GAC screening. Although traditional tumor biomarkers, such as carcinoembryonic antigen and carbohydrate antigens 19−9 and 72−4, have been widely employed in the early screening of GAC, they do not prevent the occurrence of missed diagnoses and often do not provide satisfactory predictions of treatment response [4], necessitating the exploration of emerging biomarkers. Ideal GAC biomarkers should prevent delays in diagnosis and provide insights to improve the current treatment strategies for GAC.

ATP-binding cassette A1 (ABCA1) is an integral membrane protein comprising 2261 amino acids. As a member of the ABC transporter superfamily, it can export intracellular free cholesterol and phospholipids, exerting anti-atherosclerotic effects [5, 6]. According to Wang et al., ABCA1 represents a potential therapeutic target for various cardiovascular diseases [7]. Moreover, tumor-related studies have found that ABCA1-mediated intracellular cholesterol homeostasis is altered in cancer cells, affecting membrane-anchored signaling pathways [8]. In particular, ABCA1 upregulation exacerbates the progression of colorectal cancer [9], melanoma [10], and bladder cancer [11]; ABCA1 expression is also significantly elevated in triple-negative breast cancer tissue than in normal breast tissue [5]. Moreover, while ABCA1 expression is closely associated with ovarian cancer invasion and migration [12], it exhibits anti-cancer effects in prostate cancer [13]. Meanwhile, the molecular mechanism of ABCA1 in GAC and its impact on the immune microenvironment has not been fully characterized.

Traditional RNA-sequencing evaluates the average gene expression profile across various mixed cells; however, it overlooks the data associated with specific cell subpopulations [14]. In contrast, single-cell RNA-sequencing (scRNA-seq) enables the comprehensive and multilevel analysis of heterogenous transcriptome data for tumor cells at the cell subpopulation level [15]. Indeed, while several studies have evaluated ABCA1 expression at the tumor tissue level, there is a lack of research at the single-cell level, nor is it clear how ABCA1 impacts the tumor microenvironment (TME).

In this study, bulk RNA-sequencing (bulk-seq) data from The Cancer Genome Atlas (TCGA)-Stomach Adenocarcinoma (STAD) and Gene Expression Omnibus (GEO) were used to determine the role of ABCA1 in the clinical stratification of GAC. In addition, we verified the biological characteristics of ABCA1 at the GAC cell subpopulation level based on GAC scRNA-seq data. Subsequently, the effect of ABCA1 knockdown on GAC cell invasion and proliferation was explored in vitro. Collectively, this study provides novel insights regarding the pathological mechanism of ABCA1 in GAC, which may provide new therapeutic targets for GAC.

## Materials and methods

### Data acquisition

Transcriptomic and paired clinical data containing 32 normal and 375 gastric cancer (GC) samples were obtained from TCGA database (https://cancergenome.nih.gov/), from which GC samples with incomplete clinical information were excluded. The gene expression data series profiles GSE13911 (Normal = 31, Tumor = 38), GSE27342 (Normal = 80, Tumor = 80), GSE29272 (Normal = 134, Tumor = 134), GSE54129 (Normal = 21, Tumor = 111), and GSE65801 (Normal = 32, Tumor = 32) from the GEO (http://www.ncbi.nlm.nih.gov/geo) database provided the standardized transcriptomic data of normal gastric and GC tissues. In addition, we obtained ten GC samples from GSE167297, including the scRNA-seq data from five superficial GC, five deep GC, and four paracancerous samples [16].

### ABCA1 expression analysis and diagnostic value

We assessed the differential expression of ABCA1 within GAC and normal gastric tissues in the TCGA-STAD and GEO cohorts. Participants’ receiver operating characteristic (ROC) curves were used to determine the diagnostic value of ABCA1 within the cohorts. This was followed by analyzing the relationship between ABCA1 expression and clinical features.

### Prognostic implications of ABCA1

The overall survival (OS) curves of ABCA1 in GAC samples were obtained from the Gene Expression Profiling Interactive Analysis (GEPIA) web server (http://gepia.cancer-pku.cn/index.html); the Kaplan–Meier Plotter tool (https://kmplot.com/analysis/) was employed to further verify the relationship between ABCA1 (203505_at) and OS. By consolidating the clinical information on GAC in TCGA database, univariate and multivariate Cox analyses were performed to determine whether ABCA1 was an independent prognostic factor, and the ABCA1-nomogram was constructed to predict the one-, three-, and five-year survival rates of patients with GAC.

### Functional enrichment analysis

Gene set enrichment analysis (GSEA) assists in interpreting data from gene expression profiles by setting specific functional gene sets [17]. We employed the GSEA software (v. 4.1.0) to identify the signaling pathways in which ABCA1 is involved in the TCGA-STAD cohort, with a permutation test parameter of 1000, and gene set parameters of “h.all.v2022.1.Hs.symbols.gmt” and “c2.cp.kegg.v2022.1.Hs.symbols.gmt.” The threshold for statistical significance was set at a false discovery rate (FDR) < 0.05.

### Analysis of ABCA1 and tumor-infiltrating immune cells

To assess the effect of ABCA1 on tumor-infiltrating cells (TICs), the CIBERSORT algorithm [18] was applied to analyze the abundance of TICs in each GAC sample within the TCGA-STAD dataset. Patients with GAC were then divided according to the median value of their ABCA1 expression into the high and low ABCA1 expression groups. The limma package was used to determine the difference in TIC levels between the high- and low-ABCA1 groups, and correlation analysis was performed between ABCA1 and TICs using the Spearman correlation test.

### Half maximal inhibitory concentration scores and checkpoint

To fully elucidate the predictive effect of ABCA1 expression for anti-GAC treatment, anti-tumor drug response data were obtained from the Genomics of Drug Sensitivity in Cancer (GDSC) database (https://www.cancerrxgene.org/) [19]. The pRRophetic and limma packages were then used to assess the relationship between the high- and low-ABCA1 groups and the response to different anti-tumor drugs. Subsequently, the differential expression of various immune checkpoints was compared between the high- and low-ABCA1 groups.

### Single-cell RNA-sequencing data preprocessing

The count files were read into R and formatted; the average values of duplicate genes were obtained, and the transcriptomic sequencing data of GAC cells and adjacent tissue cells were merged into one matrix. The Seurat package (v4.3.0.1) processed the scRNA-seq data for GAC and paracancerous samples [20]. Batch effects between the samples were removed using the Harmony package. The “CreateSeuratObject” function was used to process the data for each matrix, and the “merge” function was used to merge the transcriptomic matrices of different samples into one matrix. The “PercentageFeatureSet” function (parameter: pattern = “^MT-“) was used to calculate the number of mitochondrial genes and the percentage. The “subset” function (parameters: nFeature_RNA > 300; nCount_RNA > 1000; nCount_RNA < 20,000; percent.mt < 15) further filtered high-quality cells. The “NormalizeData” function (parameter: scale.factor = 10,000) was used for logarithmic normalization. The “FindVariableFeatures” function (parameters: selection.method = vst, nfeatures = 2000) identified the top 2000 highly variable genes. Subsequent principal component analysis (PCA) dimensionality reduction was performed for the 2000 highly variable genes with the “RunPCA” function (parameters: verbose = F, npcs = 50).

The t-distributed stochastic neighbor embedding (t-SNE) algorithm was used for cluster analysis. The number of cell clusters was determined through the “FindNeighbors” function (parameters: dims = 1:12) and the “FindClusters” function (parameter: resolution = 0.5). Meanwhile, the “RunTSNE” function (parameter: dims = 1:12) generated cell clusters and the “FindAllMarkers” function (parameters: min.pct = 0.3, logfc.threshold = 0.25) detected differentially expressed genes (DEGs) in each cell cluster. With the help of the CellMarker database [21], cell types were annotated, and cell subpopulations expressing ABCA1 were defined as ABCA1-related cells. The “PercentageFeatureSet” function was used to analyze the ABCA1 expression percentage in ABCA1-related cells, defining those with expression > 0 as ABCA1(+)-related cells and those with a percentage = 0 as ABCA1(−)-related cells. Subsequently, the Monocle2 package (v2.22.0) was employed to analyze the developmental trajectory of ABCA1-related cells during GAC progression [22]. The “differentialGeneTest” function was then used to identify DEGs; the “reduceDimension” function (parameters: max_components = 3, num_dim = 4, reduction_method = “DDRTree”) performed dimensionality reduction analysis, and the “orderCells” function inferred cell trajectories.

To further investigate the ABCA1-related biological mechanisms at the single-cell level in GAC, we calculated the gene clusters along the cell development trajectory and performed enrichment analysis on those with consistent ABCA1 expression patterns. The CellChat package (v1.5.0) was used to identify the interactions between ABCA1(+)-related-cells and ABCA1(−)-related-cells with different cell subpopulations [23]. Additionally, the “identifyOverExpressedGenes” function was used to identify overexpressed genes, while the “identifyOverExpressedInteractions” function identified ligand–receptor pairs. Subsequently, the “projectData” function was applied to project ligand–receptor pairs onto the protein–protein interaction (PPI) network, and the “filterCommunication” function (parameter: min.cells = 3) was used to filter cell–cell communication. Finally, the “computeCommunProbPathway” function inferred cell–cell communication, and the “aggregateNet” function computed the aggregated communication network between cells.

### Single-cell regulatory network inference and clustering analysis

Using the single-cell regulatory network inference and clustering (SCENIC) package [24], we randomly selected the gene expression profiles of 1000 ABCA1-related cells to infer the transcriptional regulatory network of ABCA1(+)-related cells. The GENIE3 function was then applied to construct the co-expression modules of transcriptional factors (TFs), and the RcisTarget package was employed to identify TFs and their regulatory targets. Evaluation of specific TFs in ABCA1(+)-related cells was performed using the regulon specificity score (RSS). In addition, we employed the AUCell package to calculate the activity of TFs in cell subpopulations and evaluated correlations between ABCA1 and specific TFs in the TIMER database (https://cistrome.shinyapps.io/timer/).

### Relationships between ABCA1-related cells and clinical phenotypes based on the Scissor algorithm

The Scissor algorithm uses phenotypic information to identify key cell subpopulations [25]; in this way, we explored the association between ABCA1-related cells and specific clinical phenotypes based on the survival status in the TCGA-STAD cohort (“Dead” and “Alive”), the “Tumor” and “Normal” phenotypes, significant indicators from previous analyses of clinical characteristics, bulk-seq data, and scRNA-seq from ABCA1-related cells. We first selected 500 ABCA1(+)-related cells with the highest ABCA1 expression and 500 ABCA1(-)-related cells with the lowest ABCA1 expression. Then, by referencing the methods described in the original paper for the Scissor algorithm [25], the clinical phenotypes were set as binary values (“0” and “1”), and the Scissor function was used to divide the ABCA1-related cells into three subpopulations: Scissor + cells, Scissor- cells, and Background cells. A phenotype with an indicator value of “1” was positively correlated with Scissor + cells, “0” was positively correlated with Scissor- cells, while Background cells were not correlated with the phenotype. Finally, a chi-square test was performed to analyze the differences in the proportions of ABCA1(+)- and ABCA1(-)-related cells for each phenotype.

### Cell culture and transfection

One normal gastric cell line (GES-1) and four GAC cell lines (MKN-45, HGC-27, BGC-823, and Hs-746T) were purchased from Hangzhou Frieden Biotechnology Co., Ltd. MKN-45, BGC-823, and HS-746T were cultured in Roswell Park Memorial Institute-1640 medium, while GES-1 and HGC-27 were cultured in Dulbecco’s Modified Eagle Medium with 10% fetal bovine serum (FBS) at 37 °C and in a 5% CO_2_ environment. Silencing of ABCA1 was achieved through the human target genes ABCA1 short hairpin RNA (shRNA; GenePharma Co., Ltd.) with the following sequences: ABCA1-shRNA1: 5′-CAATGTGGAGAGGACAAATAA-3′; ABCA1-shRNA2: 5′-CAACAGCTTGGGAAGATTTAT-3′; and ABCA1-shRNA3: 5′-CGACAAGGCCGCACCATTATT-3′. HGC-27 cells were transfected with Lipofectamine 3000 (Invitrogen) for 72 h before subsequent analyses.

### Quantitative reverse transcription PCR

To quantify ABCA1 expression in the normal gastric and GAC cell lines, total RNA was extracted from GES-1 and the four GAC cell lines using the TRIzol Reagent (Thermo Fisher, New York, USA). Quantitative reverse transcription PCR (qRT-PCR) was then performed using SYBR Premix Ex Taq (China, Chengdu Atomic Energy) according to the manufacturer’s protocol. Experiments were performed in triplicate for each group, and β-actin (ACTB) was used as the internal control. ABCA1 expression was quantified using the 2^−ΔΔCT^ method with the following primers: ACTB F5′-TGGCACCCAGCACAATGAA-3′ and R5′-CTAAGTCATAGTCCGCCTAGAAGCA-3′; ABCA1 F5′-ACCCACCCTATGAACAACATGA-3′ and R5′-GAGTCGGGTAACGGAAACAGG-3′.

### Cell proliferation assays

To evaluate the proliferative capacity of GAC cells after ABCA1 silencing, HGC-27 cells were transfected with ABCA1-shRNA and negative control (NC)-shRNA, and then incubated at 37 °C and 5% CO_2_. HGC-27 cell proliferation was evaluated using the Cell Counting Kit-8 (CCK-8, Dojindo, Tokyo, Japan); the absorbance at 450 nm was measured at 0, 24, 48, and 72 h.

### Wound healing test

The HGC-27 cells were seeded onto 6-well plates at a density of 1 × 10^5^/well. Once the cells were adherent and reached 80–100% confluence, they were scratched with a 2 µL sterile pipette tip, washed three times with phosphate-buffered saline (PBS), and cultured in fresh complete medium for 48 h. The cultures were photographed at 0, 24, and 48 h under a microscope at 100× magnification.

### Cell Invasion assays

HGC-27 cell invasion was evaluated using a Transwell chamber (Corning, New York, USA). HGC-27 cells transfected with ABCA1-shRNA or NC-shRNA were placed in the upper chamber, while complete medium was placed in the lower chamber. After incubation at 37 °C and 5% CO_2_ for 48 h, the upper layer of non-invasive cells was removed with cotton swabs, and the HGC-27 cells were fixed and stained with 4% paraformaldehyde and 0.1% crystal violet, respectively. Subsequently, the cells were observed under a microscope at 400× magnification, and analyzed using ImageJ (v1.8.0.112).

### Colony formation assays

HGC-27 cells transfected with NC-shRNA or ABCA1-shRNA were cultured for 2 weeks at a density of 500 cells/well. The cells were then fixed with 4% paraformaldehyde and stained with crystal violet, followed by quantitative analysis using ImageJ (v1.8.0.112).

### Statistical analysis

Statistical analyses were performed using R (v4.1.0). The Wilcoxon and Kruskal–Wallis tests were performed to compare differences between and across groups, respectively. The Kaplan–Meier method and log-rank test were used to determine the differences in OS between the high- and low-ABCA1 groups. Univariate and multivariate Cox regression analyses were performed to determine the independent prognostic factors. Spearman’s rank correlation was performed for correlation analysis. Graphs were plotted using R (v4.1.0) and GraphPad Prism (v7.0). All experiments were performed in triplicate. Unless stated otherwise, the differences were considered statistically significant at P < 0.05.

## Results

### ABCA1 expression and diagnostic value in GAC

**Fig. 1:**
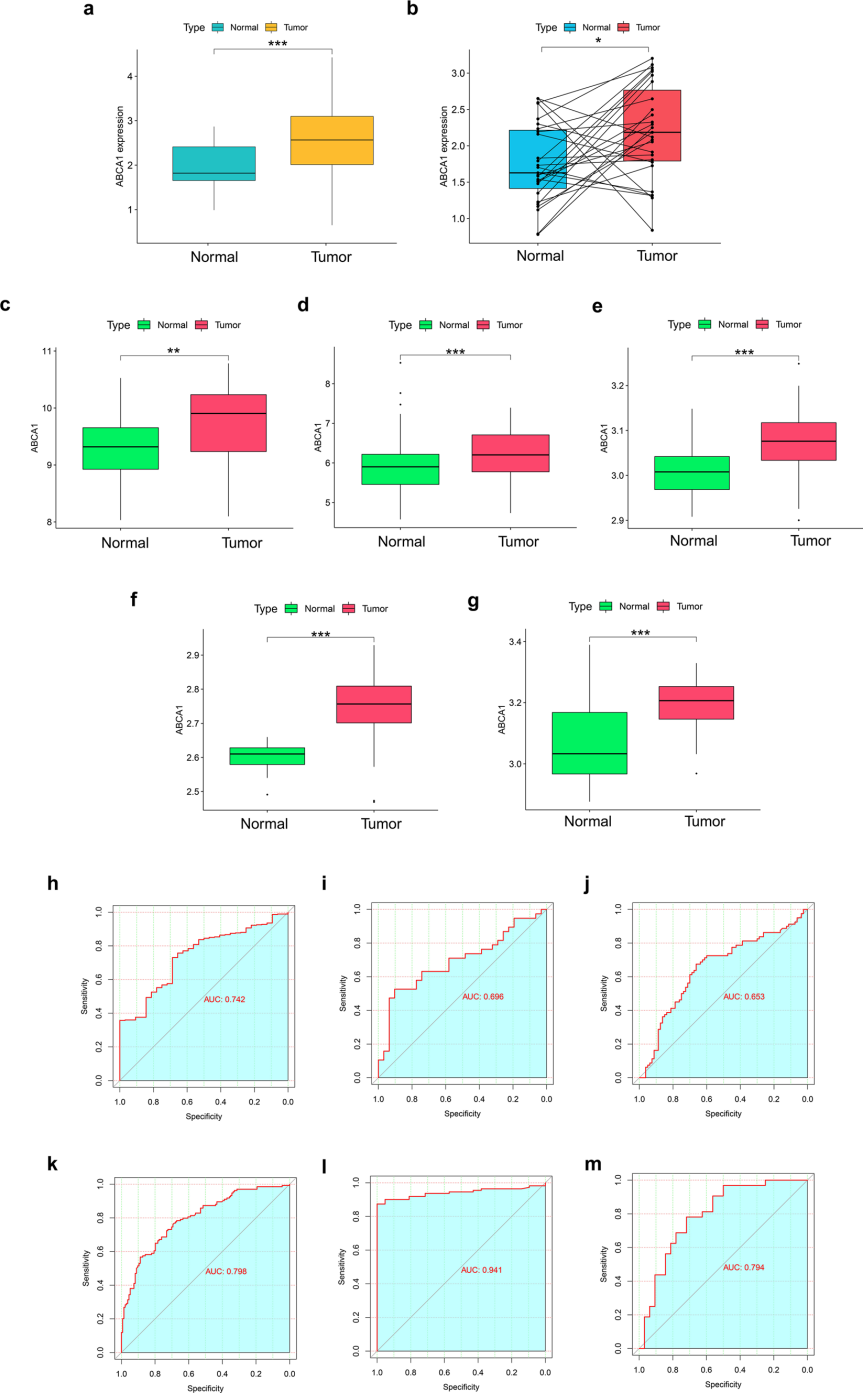
Differential expression analysis and diagnostic value of ABCA1. **a** ABCA1 differential expression in normal and GAC tissues based on TCGA-STAD cohort. **b** Differential expression of ABCA1 between GAC and paired normal tissues. **c**–**g** Differential expression of ABCA1 in the **c** GSE13911, **d** GSE27342, **e** GSE29272, **f** GSE54129, and **g** GSE65801 cohorts. **h**–**m** ROC curves for the diagnostic value of ABCA1 expression in **h** TCGA-STAD, **i** GSE13911, **j** GSE27342, **k** GSE29272, **l** GSE54129, and **m** GSE65801 cohorts

**Fig. 2:**
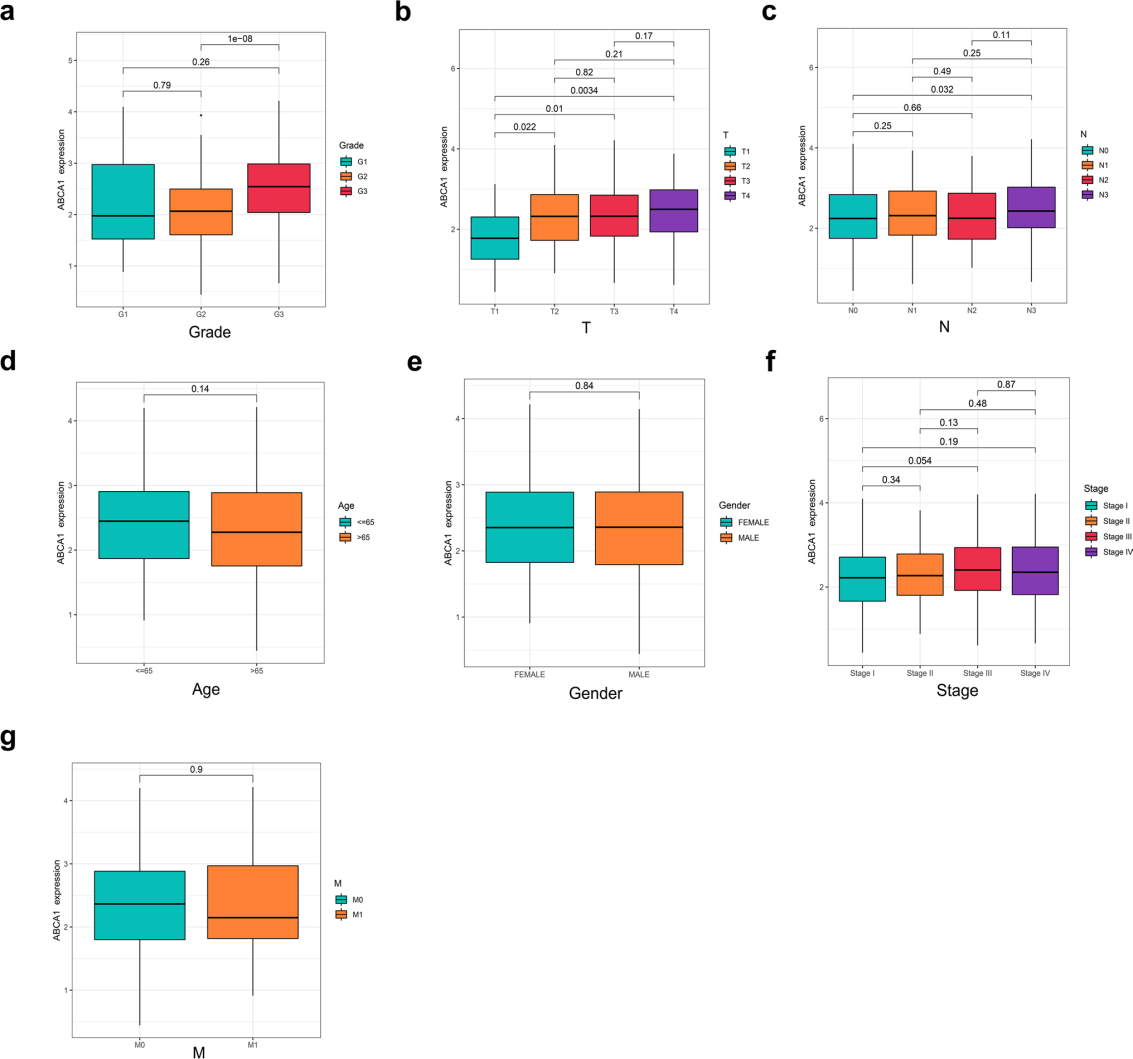
Relationship between ABCA1 expression and GAC clinical features. **a**–**g** Differential analysis of the relationship between ABCA1 expression and **a** tumor grade, **b** T stage, **c** N stage, **d** age, **e** gender, **f** TNM stage, and **g** distant metastasis

Analysis of the TCGA-STAD dataset indicated that ABCA1 expression was significantly higher in GAC tissue than in normal gastric tissue (P < 0.001; Fig. 1a) or paired normal tissue (P < 0.05; Fig. 1b). These findings were verified in the five datasets GSE13911 (P < 0.01), GSE27342 (P < 0.001), GSE29272 (P < 0.001), GSE54129 (P < 0.001), and GSE65801 (P < 0.001; Fig. 1c–g). The ROC curves further demonstrated the effectiveness of ABCA1 in discriminating between GAC and normal tissues, with the different cohorts showing areas under the curve (AUC) of 0.742, 0.696, 0.653, 0.798, 0.941, and 0.794 (Fig. 1h–m), respectively. Moreover, ABCA1 expression was elevated in high-grade GAC (P < 0.001; Fig. 2a) and was associated with the extent of tumor spread (T) and lymph node metastasis (N). ABCA1 expression was higher in the T2, T3, and T4 stages than in the T1 stage (P < 0.05; Fig. 2b), and in the N3 stage than in the N0 stage (P < 0.05; Fig. 2c). However, ABCA1 expression was not associated with age, tumor node metastasis (TNM) staging, or distant metastasis (M) (P > 0.05; Fig. 2d–g).

### Associations between ABCA1 expression and the survival prognosis of patients with GAC

**Fig. 3:**
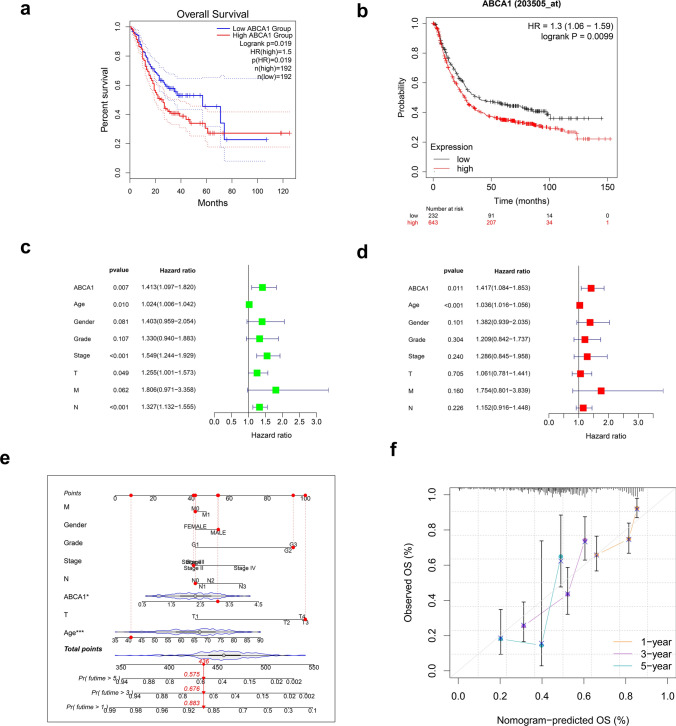
Survival analysis and prognostic value of ABCA1. **a**, **b** OS of patients with GAC with high and low ABCA1 expression in the **a** GEPIA database and **b** Kaplan–Meier Plotter tool. **c** Univariate and **d** multivariate Cox regression analysis of ABCA1 and other clinical features. **e** ABCA1-Nomogram constructed by integrating ABCA1 with various clinical features and **f** its calibration curve

To assess the prognostic value of ABCA1 in GAC, we employed the GEPIA database to perform Kaplan–Meier survival analysis. High ABCA1 expression was positively correlated with poor OS in GAC (P < 0.05; Fig. 3a). This finding was further verified using the Kaplan–Meier Plotter tool (P < 0.05; Fig. 3b). Univariate Cox analysis also suggested that high ABCA1 expression was associated with a high hazard ratio (HR) (HR = 1.413, 95% confidence interval [CI]: 1.097–1.820, P = 0.007) (Fig. 3c). The subsequent multivariate Cox analysis confirmed that ABCA1 expression (HR = 1.417, 95% CI: 1.084–1.853, P = 0.011) and age (HR = 1.036, 95% CI: 1.016–1.056, P < 0.001) were independent prognostic factors (Fig. 3d). By combining the clinical features, we constructed an ABCA1 nomogram to predict the one-, three-, and five-year survival rates of patients with GAC (Fig. 3e); the reliability of the results was confirmed via the calibration curve (Fig. 3f).

### Functional analysis of ABCA1

**Fig. 4:**
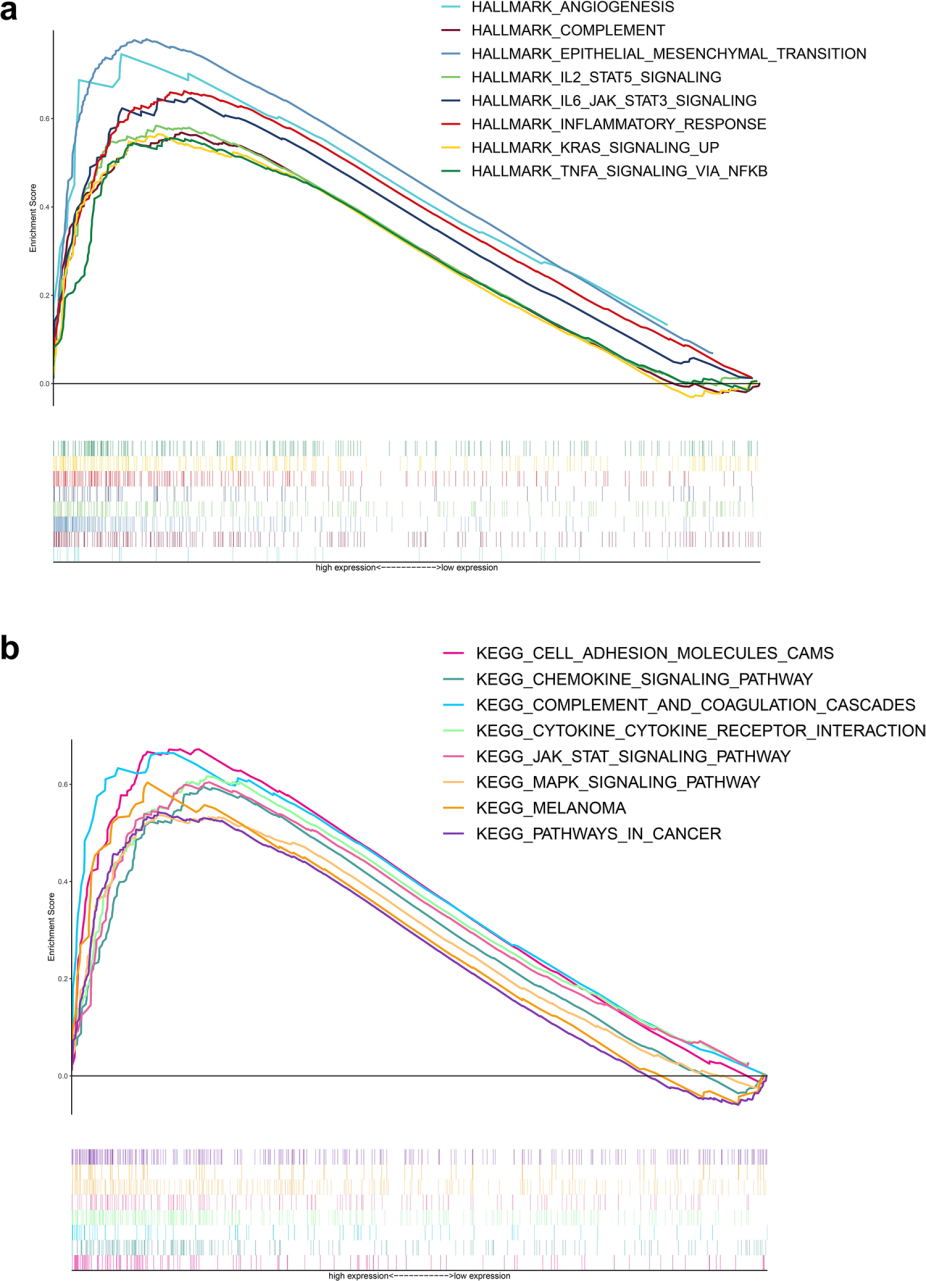
Functional enrichment analysis of ABCA1. **a**, **b** Functional enrichment analysis based on the **a** HALLMARK and **b** KEGG gene sets

Through GSEA, we explored the signaling pathways associated with ABCA1 based on two gene sets (Fig. 4a, b). The results showed that epithelial mesenchymal transition (EMT), angiogenesis, interleukin-6 (IL-6)/Janus kinase (JAK)/signal transducer and activator of transcription 3 (STAT3) signaling, inflammatory response, complement and coagulation cascades, KRAS signaling, tumor necrosis factor-alpha (TNF-⍺) signaling via nuclear factor-κB (NF-κB), and pathways in cancer were enriched in the high-ABCA1 group. Thus, high ABCA1 expression may promote the progression of GAC via these pathways.

### ABCA1 expression correlates with TICs

**Fig. 5:**
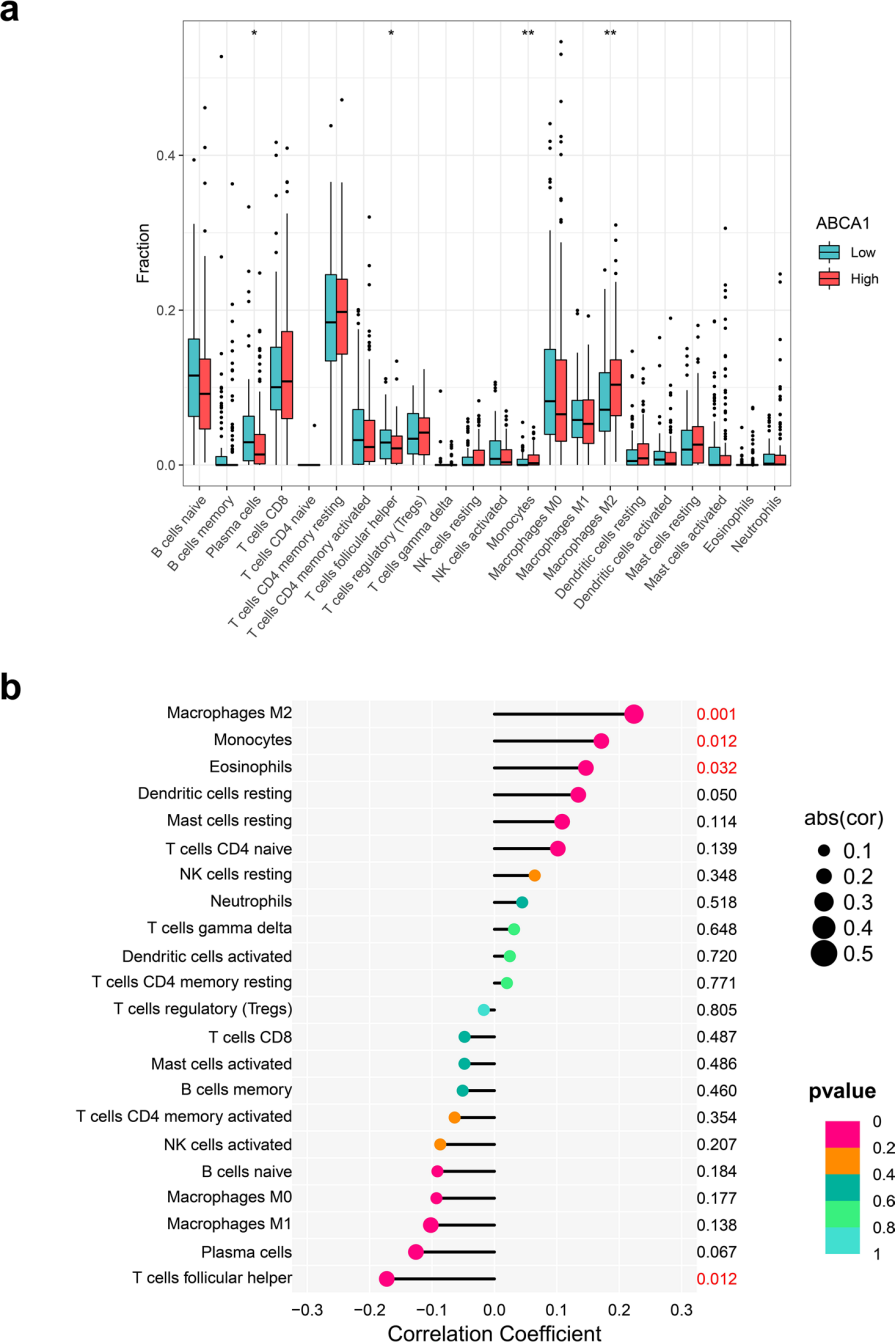
Relationships between ABCA1 expression and TICs. **a** Differences in TIC infiltration between the high- and low-ABCA1 groups. **b** Correlation analysis between ABCA1 expression and TICs.

Considering that ABCA1 might participate in immune response regulation [26], we examined the relationship between ABCA1 expression and TICs. Patients with GAC and high ABCA1 expression exhibited significantly higher infiltration of alternatively activated (M2) macrophages (P < 0.01) and monocytes (P < 0.01); M2 infiltration was positively correlated with ABCA1 expression (P = 0.001; Fig. 5a). However, the infiltration of plasma cells (P < 0.05) and T follicular helper cells (P < 0.05) was higher in the low-ABCA1 group. Additionally, ABCA1 expression was significantly positively correlated with M2 macrophage and monocyte infiltration and negatively correlated with plasma cell infiltration (P < 0.05; Fig. 5b).

### Half maximal inhibitory concentration score and checkpoint

**Fig. 6:**
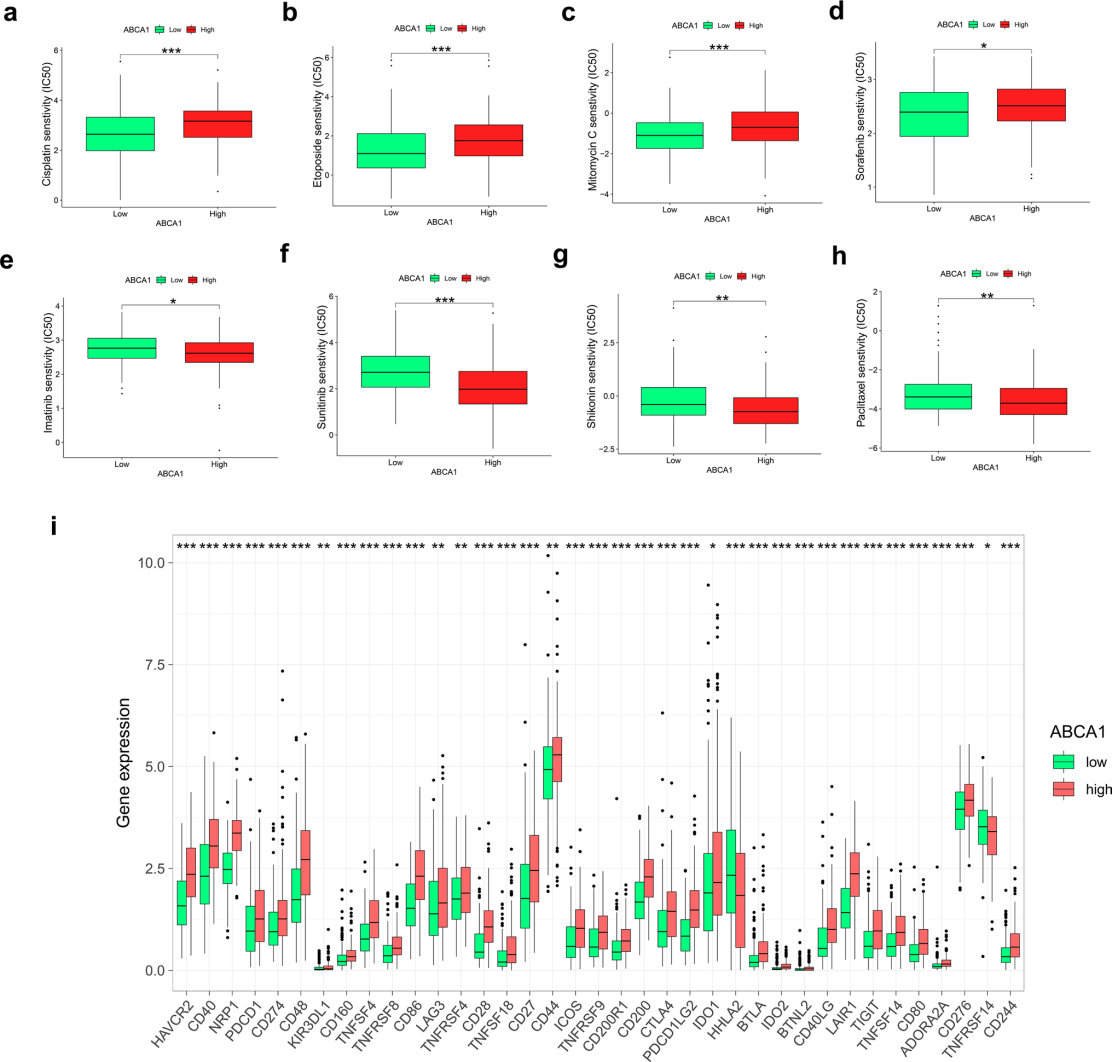
Analysis of drug sensitivity and immune checkpoints. **a** Correlation between high- and low-ABCA1 groups and anti-tumor drug sensitivity. **b** Immune checkpoint expression in the high- and low-ABCA1 groups

In the analysis of ABCA1 expression and anti-tumor drug sensitivity, we found that patients in the high-ABCA1 group had a higher half maximal inhibitory concentration (IC_50_) for cisplatin (P < 0.001), etoposide (P < 0.001), mitomycin C (P < 0.001), and sorafenib (P < 0.05) compared to patients in the low-ABCA1 group (Fig. 6a–d). In contrast, the high-ABCA1 group exhibited a lower IC_50_ for imatinib (P < 0.05), sunitinib (P < 0.001), shikonin (P < 0.01), and paclitaxel (P < 0.01) compared to the low-ABCA1 group (Fig. 6e–h). Furthermore, significantly higher expression of checkpoints, including programmed cell death protein 1 (PDCD1; P < 0.001), cytotoxic T-lymphocyte associated protein 4 (CTLA4; P < 0.001), lymphocyte-activation protein 3 (LAG3; P < 0.01), hepatitis A virus cellular receptor 2 (HAVCR2; P < 0.001), and PDCD1 ligand 2 (LG2; P < 0.001), was observed in the high-ABCA1 group (Fig. 6I).

### ABCA1 expression at the single-cell level

**Fig. 7:**
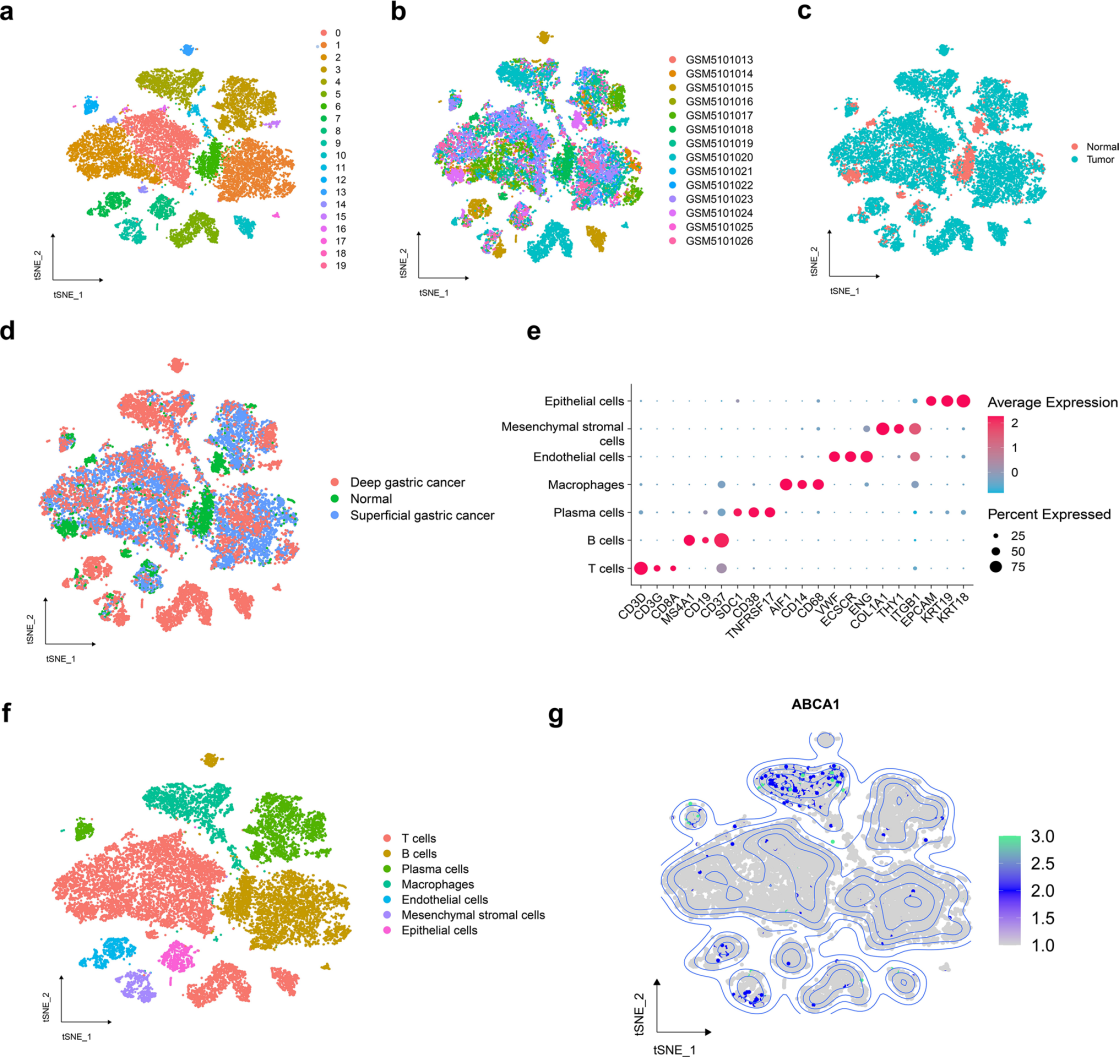
Overview of scRNA-seq data derived from ten GC and four normal gastric tissue samples. **a**–**d** t-SNE plots categorized by **a** cluster, **b** sample origin, and **c**, **d** tissue type. **e** Specific marker genes corresponding to each cell subpopulation. **f** Annotated t-SNE plot. **g** Distribution of ABCA1 expression in different cell types

From the scRNA-seq analysis of ten GAC and four paracancerous samples, we obtained 19,999 high-quality cells (Supplementary Fig. S1).The cells were subjected to dimensionality reduction through PCA. The first 12 principal components (Supplementary Fig. S2) were imported to t-SNE, and clustered into 20 subpopulations (Fig. 7a). The cell subpopulations were visualized by their sample origin (Fig. 7b) and tissue type (Fig. 7c, d), and were annotated according to specific marker genes (Fig. 7e) as T, B, plasma, endothelial, mesenchymal stromal, and epithelial cells and macrophages (Fig. 7f). ABCA1 was primarily expressed by macrophages (Fig. 7g) and in tumor tissues, not normal gastric tissues (Supplementary Fig. S3).

### Trajectory analysis

**Fig. 8:**
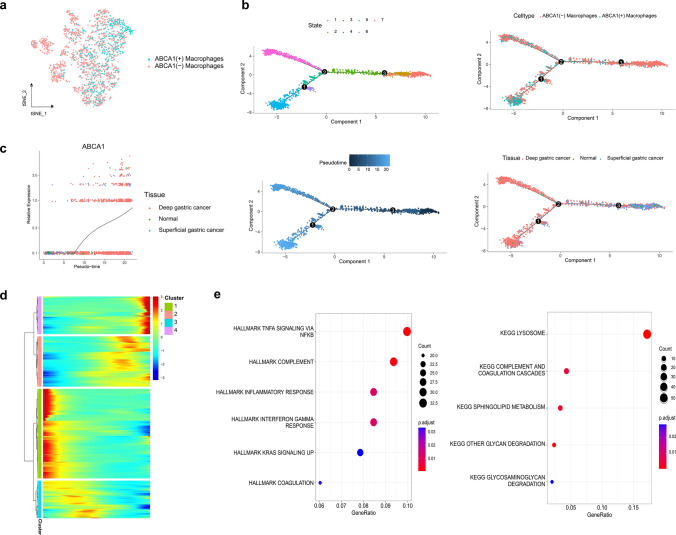
Pseudotime trajectory analysis of macrophage subpopulations. **a** Macrophages divided into the ABCA1(+) and ABCA1(−) macrophage subpopulations. **b** Trajectories of the macrophage subpopulation plotted according to state, cell type, pseudotime, and tissue. **c** Changes in ABCA1 expression with pseudotime trajectory in different tissues. **d** Dynamic gene expression in the pseudotime trajectory (red: upregulated genes; blue: downregulated genes). **e** Enrichment analysis of Cluster 4

To explore the developmental trajectory of ABCA1-related macrophages, we first isolated the macrophage subpopulations in GAC and normal gastric tissues, comprising 2082 macrophages. The PercentageFeatureSet function identified 551 ABCA1-expressing ABCA1(+) macrophages and 1531 non-ABCA1-expressing ABCA1(-) macrophages (Fig. 8a). The Monocle2 algorithm was applied to simulate the trajectory of the macrophages; macrophages were divided into seven differentiation states (Fig. 8b). Based on the pseudotime trajectory, ABCA1(+) macrophages were more enriched toward the end of the differentiation trajectory than ABCA1(−) macrophages. Macrophages derived from normal tissues and superficial GC were distributed in the area at the start of the trajectory, whereas those derived from deep GC were distributed across the middle and terminal sections. Furthermore, ABCA1 expression increased gradually with the pseudotime trajectory (Fig. 8c). The genes expressed along the macrophage differentiation trajectory were further grouped into four clusters (Fig. 8d). ABCA1 was located in Cluster 4, comprising 881 genes (Supplementary Table S1). Enrichment analysis was performed on the genes in Cluster 4, revealing pathways consistent with those previously identified by GSEA, such as TNF-⍺ signaling via NF-κB, inflammatory response, KRAS signaling, and complement and coagulation cascades (Fig. 8e). Thus, at the single-cell level, these pathways might contribute to the key mechanisms underlying the promotion of GAC pathogenesis by ABCA1 upregulation.

### Cell–cell communication analysis

**Fig. 9:**
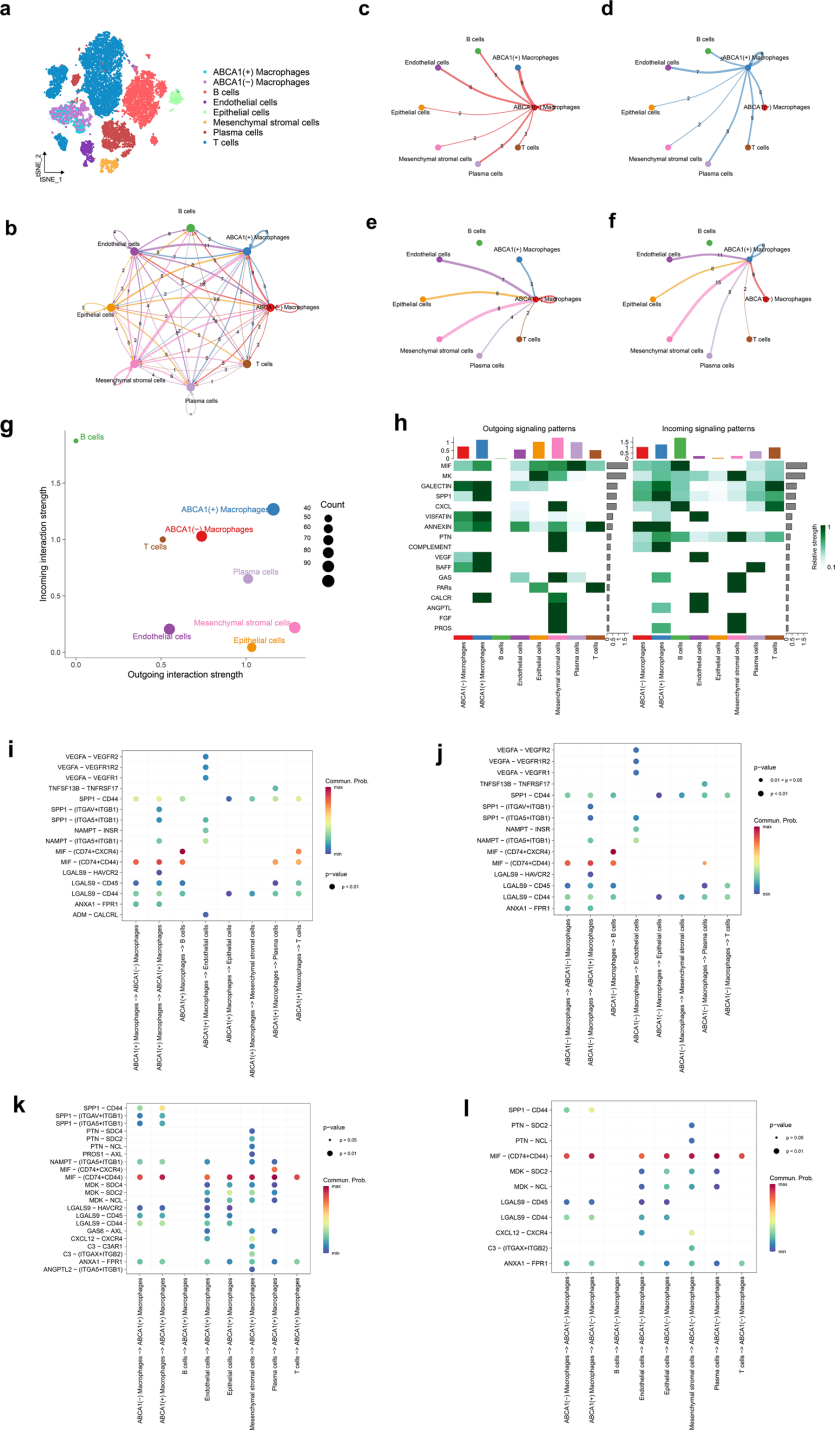
Role of ABCA1-related macrophages in cell–cell communication. **a** Macrophage subpopulations re-annotated from GAC-derived cell subpopulations. **b** Cell–cell communication between eight cell subpopulations (numbers represent the number of pathways). **c**–**f** Interactions between ABCA1(−) and ABCA1(+) macrophages with other cell subpopulations when acting as **c**, **d** signal transmitters and **e**, **f** signal receivers. **g** Strength and number of incoming and outgoing signals among cell subpopulations. **h** Heatmap demonstrating the strength of incoming and outgoing signaling pathways. **i**–**l** Ligand–receptor pairs associated with ABCA1(−) and ABCA1(+) macrophages acting as **i**, **j** signal transmitters and **k**, **l** receivers

To explore the communication among ABCA1(+) macrophages in the TME, GAC-derived cell subpopulations were extracted, and their cell types were re-annotated (Fig. 9a). Subsequently, cell–cell communication analysis was performed on eight subpopulations (Fig. 9b). When we visualized the interactions between ABCA1(−) and ABCA1(+) macrophages as signal transmitters (Fig. 9c, d) and receivers (Fig. 9e, f), the results indicated that the incoming and outgoing signals of ABCA1(+) macrophages were stronger than those of ABCA1(−) macrophages (Fig. 9g, h). Next, we explored the ligand–receptor pairs involved in the signaling pathway. When ABCA1(+) and ABCA1(−) macrophages acted as signal transmitters (Fig. 9i, j), the calcitonin receptor (CALCR) (adrenomedullin [ADM]–CALCR) pathway only appeared in the ABCA1(+) macrophage subpopulation; however, when they acted as signal receivers (Fig. 9k, l), the secreted phosphoprotein 1 (SPP1) (SPP1–(integrin subunit alpha v [ITGAV] + integrin subunit beta 1 [ITGB1]), SPP1–[ITGA5 + ITGB1]), pleiotrophin (PTN) (PTN–syndecan-4 [SDC4]), protein S (PROS) (PROS1–AXL), visfatin (nicotinamide phosphoribosyltransferase [NAMPT]–[ITGA5 + ITGB1]), macrophage migration inhibitory factor (MIF) (MIF–[CD74 + CXC chemokine receptor 4 (CXCR4)]), midkine (MK) (MDK–SDC4), galectin (LGALS9–HAVCR2), growth arrest specific (GAS) (GAS6–AXL), complement (C) (C3-C3A receptor 1 [C3AR1]), and angiopoietin-like (ANGPTL) (ANGPTL2–[ITGA5 + ITGB1]) pathways were only enriched in the ABCA1(+) macrophage subpopulation. Therefore, we speculate that ABCA1 overexpression in tumor-associated macrophages may mediate the tumor-promoting effects of these signaling pathways within the TME.

### Transcriptional regulatory network of ABCA1(+) macrophages

**Fig. 10:**
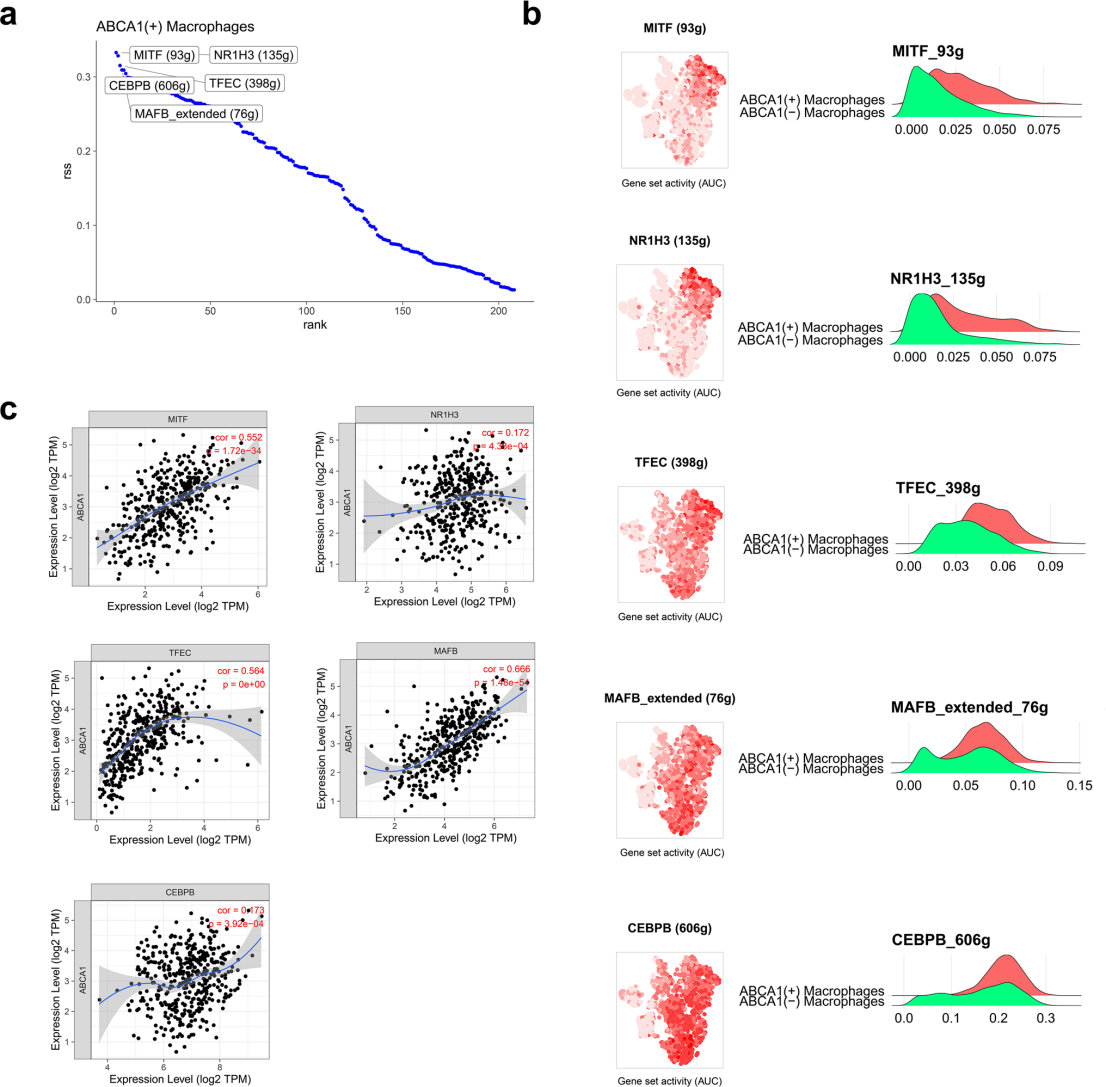
Transcriptional regulation of ABCA1 in GAC. **a** Top five TFs with the highest specificity among ABCA1(+) macrophages. **b** Activity of MITF, NR1H3, TFEC, MAFB, and CEBPB in the macrophage subpopulations. **c** Correlation analysis of MITF, NR1H3, TFEC, MAFB, and CEBPB with ABCA1.

The SCENIC algorithm was applied to analyze the specific TFs in ABCA1(+) macrophages. Based on the RSS, melanocyte inducing transcription factor (MITF), nuclear receptor subfamily 1 group H member 3 (NR1H3), transcription factor EC (TFEC), mAF BZIP transcription factor B (MAFB), and CCAAT/enhancer binding protein (C/EBP), beta (CEBPB) were identified as the five TFs with the highest specificity in the ABCA1(+) macrophage subpopulation (Fig. 10a). These TFs were more active in the ABCA1(+) macrophage subpopulation (Fig. 10b) and were positively correlated with ABCA1 expression (P < 0.05; Fig. 10c). Therefore, we speculate that ABCA1(+) macrophages were subjected to the regulation of these TFs, promoting the formation of an adverse TME.

### 10 Scissor algorithm identifies the relationship between ABCA1(+) macrophages, tumor phenotype, and survival rate

**Fig. 11:**
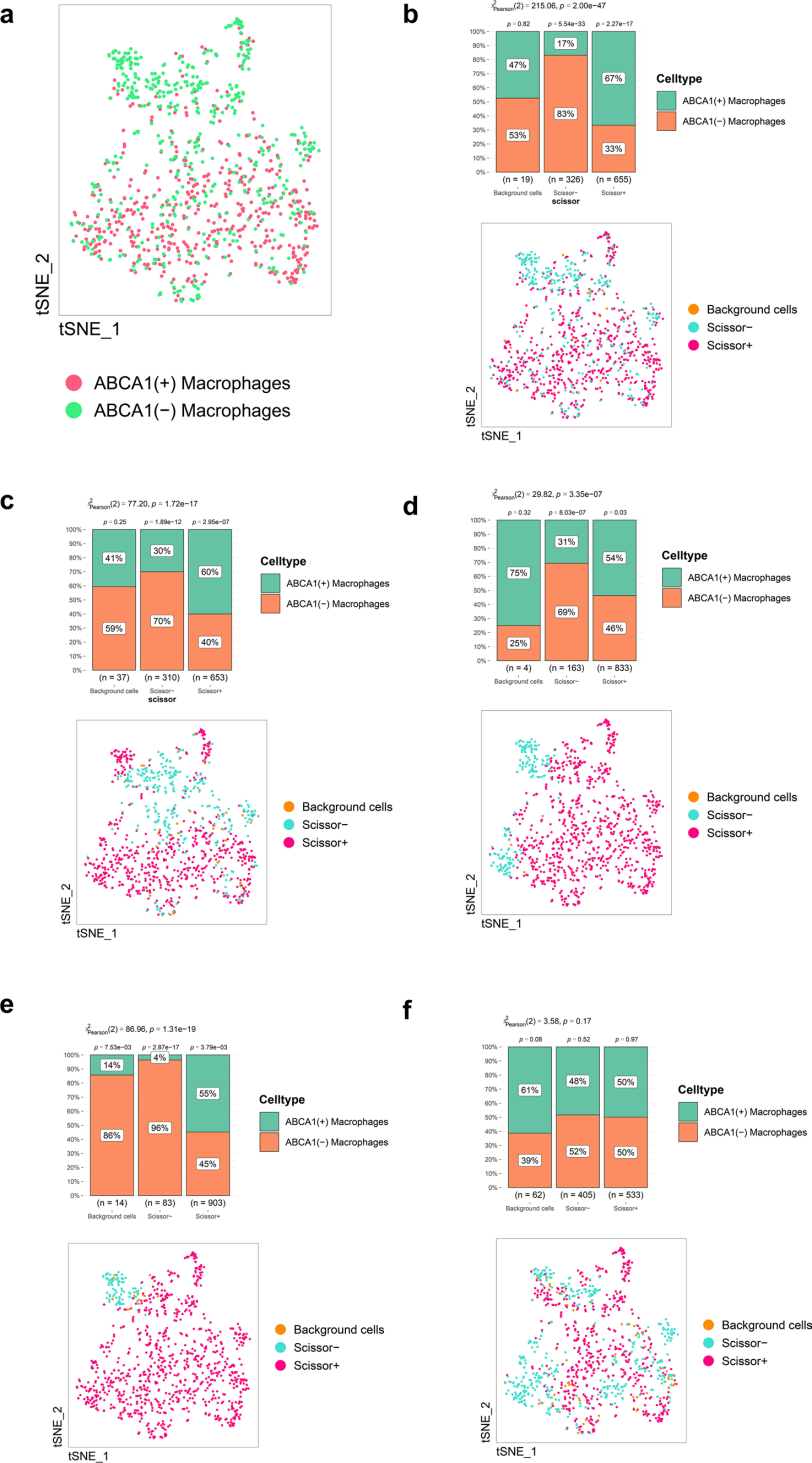
Scissor analysis of ABCA1-related macrophages. **a** The Scissor analysis comprised 1000 macrophages. **b**–**f** Relationship between ABCA1(+) and ABCA1(−) macrophages and **b** survival status, **c** Tumor vs. Normal, **d** grade, **e** N stage, and **f** T stage

During analysis of the clinical features, we found that ABCA1 expression was significantly correlated with grade, T stage, and N stage. Therefore, the phenotype indicator values of the “Dead,” “Tumor,” “Grade 3,” “T3–4,” and “N1–3” were set as “1,” while those of “Alive,” “Normal,” “Grade 1–2,” “T1–2,” and “N0” phenotypes were set as “0.” As described in the methods, 500 ABCA1(+) macrophages and 500 ABCA1(−) macrophages were selected (Fig. 11a), from which the number of Scissor + cells, Scissor- cells, and Background cells were enumerated for each clinical feature. Compared to ABCA1(−) macrophages, ABCA1(+) macrophages accounted for a higher proportion of Scissor + cells with the “Dead,” “Tumor,” “Grade 3,” and “N1–3” phenotypes (P < 0.05); ABCA1(−) macrophages accounted for a higher proportion of Scissor- cells with the “Alive,” “Normal,” “Grade 1–2,” “T1–2,” and “N0” phenotypes (P < 0.05; Fig. 11b–f). Notably, there was no significant difference in the distribution of ABCA1(+) and ABCA1(−) macrophages among T stages (Fig. 11f; P > 0.05). Our findings suggested that ABCA1(+) macrophages were positively correlated with adverse clinical phenotypes in patients with GAC.

### Knockdown of ABCA1 inhibits GAC cell growth and metastasis in vitro

**Fig. 12:**
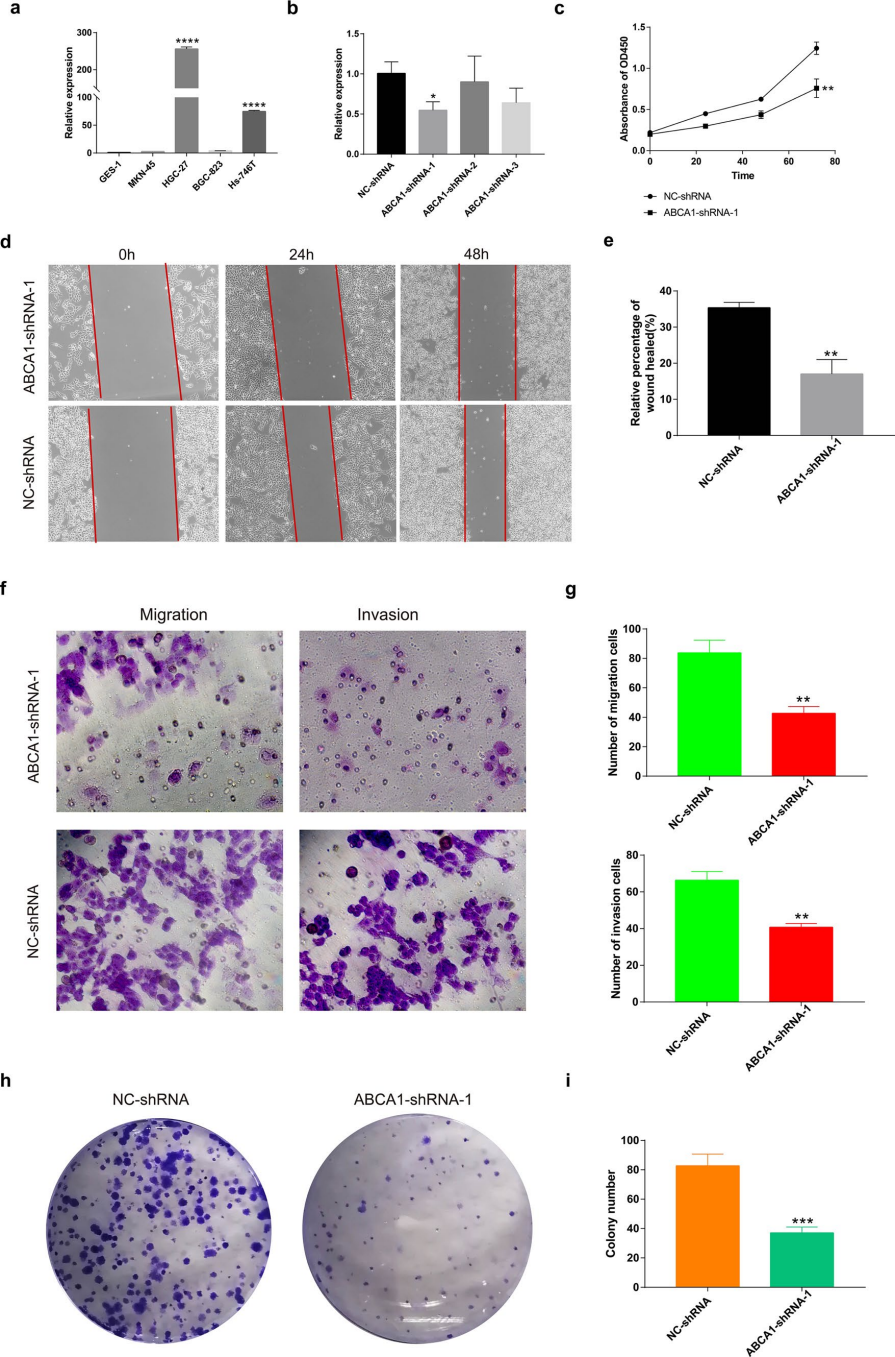
Effect of ABCA1 knockdown on GAC cell proliferation and invasion. **a** qRT-PCR analysis of ABCA1 differential expression in normal gastric cells and GAC cells. **b** The downregulation of ABCA1-shRNA-1 was most significant in HGC-27 cells. **c** Proliferation and viability of HGC-27 cells after ABCA1 knockdown. **d**, **e** Migration ability of HGC-27 cells after ABCA1 knockdown. **f**, **g** Invasion and migration abilities of HGC-27 cells between the NC-shRNA and ABCA1-shRNA groups. **h**, **i** Number of HGC-27 clones between the NC-shRNA and ABCA1-shRNA groups

The effects of ABCA1 expression on the proliferation and migration of GAC cells was evaluated in vitro. The qRT-PCR analysis showed that the ABCA1 expression was significantly upregulated in GAC cells (HGC-27 and HS-746T) compared with that in normal gastric cells (GES-1), with the most significant expression being observed in HGC-27 cells (Fig. 12a). Thus, HGC-27 cells were selected for subsequent functional experimental analyses.

Three types of shRNA were used to suppress ABCA1 expression in HGC-27 cells. The qRT-PCR assay revealed that ABCA1-shRNA-1 had the best silencing efficiency (Fig. 12b), and hence was used in subsequent experiments.

The results of the CCK8 assay showed that following ABCA1 knockdown, the abundance of HGC-27 cells significantly decreased compared to the NC-shRNA group (Fig. 12c). The wound healing assay further revealed that ABCA1-knockdown GAC cells exhibited attenuated healing ability across the different time points (Fig. 12d, e). The Transwell assay showed that the migration and invasion abilities of HGC-27 cells were significantly reduced after ABCA1 knockdown (Fig. 12f, g). Finally, the colony formation assay showed that ABCA1 silencing significantly reduced the number of new HGC-27 clones (Fig. 12h, i).

## Discussion

ABCA1 is involved in the progression of various cancers and pathological biological processes, including dyslipidemia, neurological disorders, glaucoma, and diabetes [8] However, the effect of ABCA1 on the heterogeneous TME of GAC has not been fully elucidated. Nevertheless, Liu et al. employed bioinformatics techniques to develop a lipid droplet metabolism model for GAC and revealed that upregulated ABCA1 might contribute to the development of unfavorable clinical characteristics in GAC [27]. Similarly, Yin et al. discovered a correlation between ABCA1 expression and drug metabolism. They also developed a predictive prognostic model for patients with GAC related to drug metabolism [28]. However, these previous studies concentrated primarily on bulk RNA-seq data without delving into the pathogenic processes of ABCA1 using scRNA-seq. Furthermore, these studies failed to verify the effect of ABCA1 in GAC through in vitro assays. In contrast, the current study combined bulk RNA-seq, scRNA-seq, and in vitro studies to investigate the putative oncogenic pathways of ABCA1 in GAC. As such, the findings of this study contribute to the establishment of a theoretical foundation for future targeted therapies utilizing ABCA1 in the treatment of GAC.

First, we analyzed the GAC bulk-seq data in TCGA-STAD and five GEO cohorts. The results revealed that ABCA1 was highly expressed in GAC than in normal gastric tissue, while ROC curves confirmed that it had good diagnostic value, suggesting that ABCA1 may function as an oncogene in GAC. This differential expression in ABCA1 was further verified by in vitro experiments. High ABCA1 expression was generally associated with adverse clinical features and poor OS in GAC. Additionally, multivariate Cox analysis suggested that ABCA1 was an independent risk factor. Therefore, to enhance the application value of ABCA1 in the screening of high-risk patients with GAC, we constructed an ABCA1-nomogram for the accurate and reliable prediction of OS among patients with GAC.

In addition, GSEA was performed to elucidate the potential mechanisms underlying the oncogenic functions of ABCA1. Previous studies have demonstrated that EMT activation, angiogenesis, and IL-6/JAK/STAT3 pathways increase the risk of tumor invasion, metastasis, and drug resistance [29–31]. For instance, Prijic et al. revealed that EMT regulates ABCA1 expression in breast cancer cells [32]. Meanwhile, Fang et al. found that ABCA1 knockdown leads to dysregulated angiogenesis in zebrafish [33]. In another study, the overactivation of the IL-6/JAK/STAT3 pathway was found to promote tumor proliferation and invasion while suppressing anti-tumor immune responses [30]. Frisdal et al. observed that IL-6 can stimulate ABCA1 expression by activating the JAK-STAT3 pathway, regulating macrophage cholesterol homeostasis [34]. Our findings also indicate that the complement, inflammatory response, KRAS signaling, and TNF-⍺ signaling via NF-κB pathways were activated in patients with GAC and high ABCA1 expression. Magrini et al. reported that complement activation not only induces the formation of an inflammatory TME and suppresses anti-tumor immunity, but also promotes various processes, such as tumor angiogenesis and EMT, accelerating the invasion and migration of tumor cells [35]. The important role of the KRAS signaling and TNF⍺ signaling via NF-κB pathways in tumor progression has garnered significant attention [36, 37]. However, further investigation is needed with a focus on the relationship between ABCA1 and these pathways in GAC.

The TME is a complex and heterogenous environment characterized by hypoxia, low pH, and numerous growth factors and proteolytic enzymes. It also contains myriad tumor cells, immune cells, and interstitial tissues [38]. Our study demonstrated that ABCA1 upregulation impacted the infiltration of TICs in the GAC TME, especially with respect to the increased recruitment of M2 macrophages. M2 macrophages are associated with the elevated expression of factors that promote angiogenesis and stimulate tumor cell proliferation in vivo, including matrix metalloproteinases, IL-10, and vascular endothelial growth factor [39]. Indeed, Guo et al. reported that M2 macrophages facilitate cancer cell invasion by promoting EMT and αB-crystallin expression [40]. Furthermore, ABCA1 expression negatively correlated with plasma cells and T helper cells; plasma cells have a positive role in anti-tumor immunity [41]. Meanwhile, T helper cells secrete various cytokines, including IL-2, TNF-⍺, and IL-12, which enhance natural killer cell-mediated immune responses to exert anti-tumor effects [42]. This aligns with apparent immune response suppression elicited by ABCA1 overexpression in GAC.

Drug resistance is a major constraint in the application of chemotherapy. Therefore, exploring biomarkers that can predict the sensitivity to anti-tumor drugs has significant implications for improving the outcome of precision treatment and prolonging the OS of patients with GAC [43]. We found that patients with high ABCA1 expression were more likely to exhibit resistance to cisplatin, etoposide, mitomycin C, and sorafenib, and were more sensitive to imatinib, sunitinib, shikonin, and paclitaxel. Prior research has elucidated the underlying mechanism by which ABCA1 modulates the sensitivity of malignancies to cisplatin. For instance, Chen et al. revealed that reduced ABCA1 expression can augment the susceptibility of lung cancer cells toward cisplatin through a mechanism governed primarily by Valproic acid [44]. Furthermore, Wang et al. found that ANXA2 overexpression facilitates the emergence of resistance to etoposide in neuroblastoma by activating the NF-κB signaling pathway [45]. Indeed, a positive association has been reported between mitomycin C resistance in bladder cancer and activation of the EMT and NF-κB signaling pathways [46]. In conjunction with the enrichment analysis conducted in the current study, it was observed that the EMT and NF-κB pathways exhibited frequent enrichment in GAC patients with high ABCA1 expression. This finding suggests that ABCA1 overexpression may serve as an underlying mechanism contributing to the development of resistance against etoposide and mitomycin C. In addition, ABCA1 overexpression could facilitate the activation of the IL6/JAK/STAT3 signaling pathway. Similarly, Jiang et al. reported that IL-6 activation in liver cancer leads to an increase in STAT3 expression, resulting in heightened resistance to sorafenib treatment. Moreover, Leporini et al. evaluated the use of imatinib as an anti-angiogenic modulator in patients with GAC and bone metastases [47]. Meanwhile, Hojo et al. reported that combined sunitinib and pterostilbene therapy exerts significant anti-tumor effects in GAC [48]. The combination of sunitinib with S-1 and cisplatin has also been applied in treating GAC [49]. Additionally, shikonin reportedly suppresses the invasion and activity of GAC cells; its underlying mechanism may be related to the Toll-like receptor 2/NF-κB-mediated pathway, and reactive oxygen species [50, 51]. In contrast, paclitaxel acts on tubulin in cancer cells, promoting microtubule polymerization and leading to cell division suppression [52]. It has also exhibited good therapeutic efficacy in GAC in several clinical trials [53–55]. However, further research is needed to elucidate the drug resistance mechanism of ABCA1 in GAC.

Recently, immunotherapy has fundamentally altered the direction of tumor treatment; PDCD1/PDCD1 ligand 1 (PDCD1LG1) drugs have been applied in first-line clinical trials for GAC [56, 57]. However, the application of immunotherapy is predicated on selecting clinically beneficial groups. Our study revealed that patients with GAC and high ABCA1 expression exhibited upregulation of immune checkpoints, such as PDCD1, CTLA4, LAG3, HAVCR2, and PDCD1LG2, suggesting that adjustments may be needed to the immunotherapy strategies of patients with high ABCA1 expression to achieve beneficial therapeutic outcomes.

To our knowledge, this study is the first to identify the ABCA1 features of GAC at the single-cell level. Based on scRNA-seq data, we confirmed ABCA1 expression in the T cell, plasma cell, endothelial cell, mesenchymal stromal cell, B cell, macrophage, and epithelial cell subpopulations, among which, macrophages exhibited the highest expression. However, ABCA1 expression was negligible in the cells of normal gastric tissues. Similarly, Hoppstädter et al. observed that, compared with normal lung tissues, the elevated ABCA1 expression in tumor-associated macrophages of lung adenocarcinoma promotes cholesterol efflux from macrophages [58]. A similar phenomenon was observed in a mouse model of ovarian cancer, wherein the cholesterol efflux mediated by ABCA1 upregulation promoted the transition of macrophages to M2 macrophages [59]. The exported cholesterol may support tumor cell growth [59, 60]. In the current study, cell trajectory analysis demonstrated that ABCA1(+) macrophages appeared primarily toward the end of the differentiation trajectory, consistent with the differentiation direction of GAC cells from the superficial to deep layers. Moreover, ABCA1 expression increased with the progression of GAC infiltration, implying that ABCA1 upregulation may promote the onset and development of GAC. Furthermore, the expression pattern of genes in Cluster 4 was consistent with that of ABCA1, and enrichment analysis revealed that they were involved primarily in TNF-⍺ signaling via NF-κB, complement, inflammatory response, KRAS signaling, and complement and coagulation cascade pathways. These findings agree with the bulk-seq data-based GSEA, suggesting that these pathways represent the key biological mechanisms for promoting GAC invasion and metastasis by ABCA1(+) macrophages.

Cell–cell communication analysis revealed significant differences between the cell communication networks of ABCA1(+) and ABCA1(−) macrophages. That is, when the macrophages acted as signal transmitters, the CALCR (ADM-CALCRL) pathway was only enriched in the ABCA1(+) macrophage subpopulation. However, when the macrophages acted as signal receivers, significant differences were observed between the communication networks of ABCA1(+) and ABCA1(−) macrophages. More specifically, the SPP1 (SPP1–[ITGAV + ITGB1], SPP1–[ITGA5 + ITGB1]), PTN (PTN–SDC4), PROS (PROS1–AXL), visfatin (NAMPT–[ITGA5 + ITGB1]), MIF (MIF–[CD74 + CXCR4]), MK (MDK–SDC4), galectin (LGALS9–HAVCR2), GAS (GAS6–AXL), complement (C3–C3AR1), and ANGPTL (ANGPTL2–[ITGA5 + ITGB1]) pathways were significantly enriched in the ABCA1(+) macrophage subpopulation. The ligand–receptor pairs in the CALCR, SPP1, PROS, MIF, MK, galectin, GAS, and complement pathways are closely associated with tumorigenesis, while ADM can promote the angiogenic effect of epithelial cells by binding to the CALCRL receptor [61]. Larrue et al. found that activation of the ADM-CALCRL axis in acute myeloid leukemia predicts an unfavorable prognosis and is associated with chemoresistance [62]. Additionally, SPP1 and ITGB1 participate in GAC chemoresistance via the ITGB1/YBX1/SPP1/NF-κB pathway [63]. Meanwhile, in a fibrotic microenvironment, the binding of SPP1 to ITGAV inhibits the apoptosis of lung cancer cells [64]. Further, Zhu et al. demonstrated that ITGA5 overexpression promotes poor prognosis in GAC [65]. In the PROS pathway, the PROS1 ligand and its receptor AXL are overexpressed in thyroid cancer, while AXL blockade suppresses the invasion of thyroid cancer cells [66]. Within the TME, the tumor homing of mesenchymal stromal cells is regulated by the MIF-(CD74 + CXCR4) ligand–receptor pair in the MIF pathway [67]. The MDK-SDC4 ligand–receptor pair mediates the interaction between fibroblasts and ovarian cancer cells, and is closely associated with the survival prognosis of ovarian cancer [68]. In addition, the LGALS9-HAVCR2 ligand–receptor pair is an important target for mediating immune escape in various cancers, including leukemia, breast cancer, and melanoma [69, 70]. Extensive research has also been conducted on the GAS signaling pathway in cancer. For example, Zhu et al. reported that the GAS6-AXL axis promotes tumor cell invasion, drug resistance, and mitosis by activating downstream pathways, such as the PI3K-AKT-mTOR and NF-κB pathways [71]. Furthermore, the complement system is often dysregulated in the TME. Lawal et al. found that C3-C3AR1 is involved in immune escape associated with dysfunctional T cell phenotypes [72]. However, little is known regarding the role of the PTN (PTN–SDC4), visfatin (NAMPT–[ITGA5 + ITGB1]), MK (MDK–SDC4), and ANGPTL (ANGPTL2–[ITGA5 + ITGB1]) ligand–receptor pairs in tumors; this requires further investigation. In summary, the ABCA1(+) macrophage subpopulation may accelerate the progression of GAC via signaling pathways mediated by these ligand–receptor pairs.

The dysregulation of TFs greatly increases the risk of tumor progression. Therefore, drug targets tailored to TFs have been widely employed in cancer therapy [73]. In this study, the SCENIC algorithm was employed to identify the five main TFs specific to the ABCA1(+) macrophage subpopulation (NR1H3, MITF, TFEC, MAFB, and CEBPB). Wang et al. reported that microRNA 585, CAMP-responsive element binding protein 1, and mitogen-activated protein kinase 1 inhibit GAC cell proliferation and invasion by downregulating MITF [74]. In contrast, NR1H3 upregulation activates hypoxia-induced EMT, reduces the survival rate of GAC patients [75], and promotes the metastasis of renal cell carcinoma by regulating NOD-, LRR-, and pyrin domain-containing protein 3 inflammasomes [76]. TFEC dysregulation is also associated with the progression of various tumors [77]. For example, Samir et al. demonstrated that MAFB serves as a biomarker of poor prognosis in lung adenocarcinoma [78]. In GAC, CEBPB upregulation enhances the proliferative activity of cancer cells [79]. Moreover, using the TIMER database, we confirmed that these five TFs were significantly positively correlated with ABCA1 expression. Taken together, these findings provide new insights into the molecular mechanisms underlying the ABCA1 promotion of GAC progression.

The Scissor algorithm was used to identify cell subpopulations associated with known phenotypes [26]. Combined with the clinical phenotypes in TCGA-STAD cohort, we found that Scissor + cells associated with the “Dead,” “Tumor,” “Grade 3,” and “N1–3” phenotypes exhibited a higher distribution of ABCA1(+) macrophages. Conversely, ABCA1(−) macrophages were primarily distributed in Scissor- cells associated with the “Alive,” “Normal,” “Grade 1–2,” and “N0” phenotypes. This suggests that infiltration of the ABCA1(+) macrophage subpopulation in the TME may exacerbate GAC progression and poor prognosis.

ABCA1 silencing significantly inhibits the migration and invasion of ovarian cancer cells [13]. Similarly, our study revealed that ABCA1 expression was significantly higher in GAC cells than in normal gastric cells, and was highest in the HGC-27 cell line. Furthermore, ABCA1 knockdown significantly impeded the proliferation, invasion, and migration of HGC-27 cells.

The clinical importance of anti-cancer medications that specifically target the metabolic processes of cholesterol has been demonstrated. In particular, ABCA1 plays a significant role in the context of cellular cholesterol excretion processes. The regulation of intracellular cholesterol levels by ABCA1 has an indirect impact on the proliferation, migration, and invasion of cancer cells [80–82]. In particular, lipid rafts, which are abundant in cholesterol, have a significant impact on the adhesion and migration of cancer cells [83, 84]. Moreover, the activity of phosphoinositide 3-kinase exhibits a regulatory effect on the expression of surface proteins, including ABCA1. This regulatory mechanism has been associated with a notable increase in the likelihood of cancer cells entering the circulation and subsequent metastases [85]. Moreover, as a result of the intricately orchestrated sequence of oncogenic gene mutations in cancer, which give rise to diverse metabolic modifications, ABCA1 has been recognized as a gene that exhibits a synergistic response [86]. That is, molecules in traditional signaling pathways are associated with the action of ABCA1. In their study, Wang et al. discovered that activation of the ERK/c-Jun pathway by nicotinamide N-methyltransferase increases ABCA1 expression. This, in turn, reduces cholesterol levels within cancer cells, facilitating invasion and migration in the context of triple-negative breast cancer. Moreover, ABCA1 overexpression induced by nicotinamide N-methyltransferase is a notable factor that drives EMT [87]. Similarly, the pathway involving ERK/Fra-1/ZEB1 is responsible for promoting thyroid cancer cell invasion and progression through EMT, facilitated by ABCA1 [88]. Furthermore, Prijic et al. revealed that the upregulation of ABCA1 expression in breast cancer cell line EMT is mediated by MYC by eliminating its proximal E-box elements [30]. The current study showed an enrichment of EMT pathways in patients with GAC and high ABCA1 expression. This aligns with the outcomes of prior research studies. Indeed, the close relationship between the EMT pathway and the unfavorable evolution of malignant tumors involving ABCA1 is evident. Accordingly, we hypothesize that the modulation of ABCA1 could represent a significant advancement in treating GAC by regulating EMT or pathways associated with cholesterol metabolism. Nevertheless, the precise mechanisms underlying the effects of ABCA1 remain uncertain, necessitating additional investigations into its role in the onset, progression, infiltration, and dissemination of GAC. Furthermore, the existing body of research pertaining to the therapeutic targeting of ABCA1 in tumor treatment is limited, with a dearth of pertinent clinical trials that substantiate the potential benefits of ABCA1 targeting, specifically in terms of progression-free survival and OS. Accordingly, clinical trials are underway to assess the efficacy of ABCA1-targeted treatment in malignant tumors across different cancer types.

The present study has certain limitations. First, we primarily investigated the biological impacts of ABCA1 by utilizing publicly accessible data and in vitro cell studies. Hence, further in vivo trials are needed, as well as theoretical studies on the upstream and downstream signaling pathways that mediate ABCA1. Second, it is imperative to validate the prognostic significance of ABCA1 in GAC through extensive clinical investigations involving a large cohort of patients with GAC.

This work aims to provide a comprehensive understanding of the association between ABCA1 and macrophages in GAC, shedding light on the significance of ABCA1(+) macrophages within the GAC TME. Moreover, we contend that investigating the involvement of ABCA1(+) macrophages through scRNA-seq technology across various subtypes and stages of GAC will serve as a prospective avenue for future research.

## Conclusion

ABCA1 exerts tumor-promoting effects in GAC, with its high expression associated with malignant phenotypes and poor prognosis. Analysis of scRNA-seq data showed that as a biomarker of GAC-associated macrophages, ABCA1 can induce the formation of an unfavorable TME, while in vitro studies confirmed that ABCA1 knockdown suppresses GAC progression. By comprehensively decoding the role of ABCA1 in GAC, our findings provide a potential target for the individualized treatment of GAC to improve patient outcomes.

### Supplementary Information

Supplementary material 1

Supplementary material 2

## Data Availability

The original contributions presented in the study are included in the article/Supplementary Material. Further inquiries can be directed to the corresponding author.
