# Supplementary material for: Integrated analysis of single-cell and bulk RNA-sequencing reveals the poor prognostic value of ABCA1 in gastric adenocarcinoma
Source: Discov Oncol. 2023 Oct 24;14:189. doi: 10.1007/s12672-023-00807-y (PMC10597929; doi:10.1007/s12672-023-00807-y)
Supplement: Supplementary file 1 — Supplementary material 1 [file 12672_2023_807_MOESM1_ESM.pdf]

Supplementary Material

Supplementary Figures

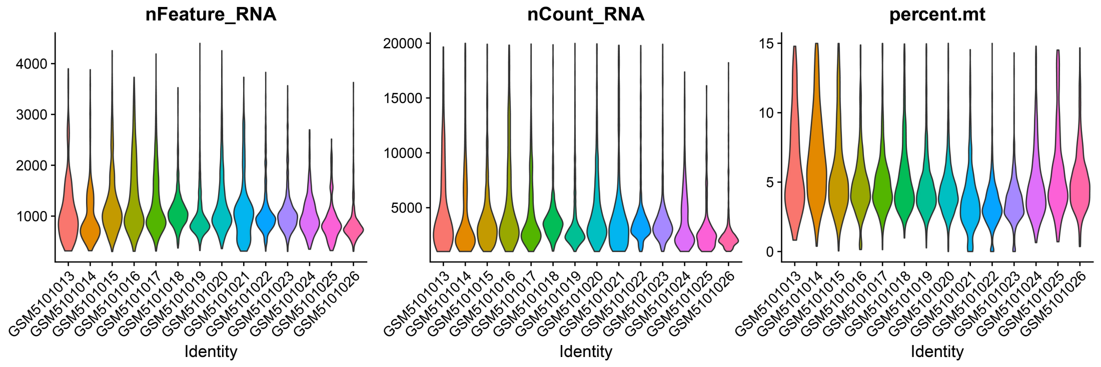

Supplementary Fig. S1 Quality control of scRNA-seq data.

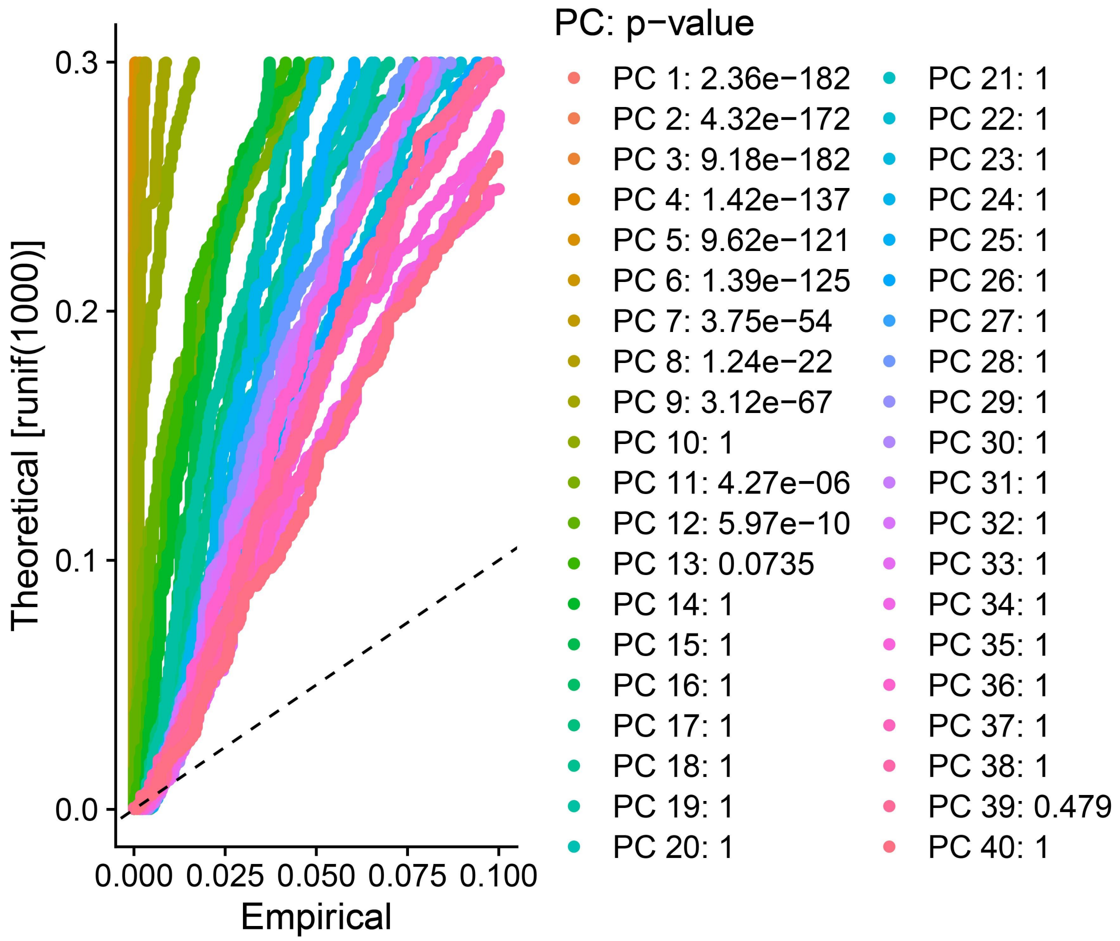

Supplementary Fig. S2 First nine principal components obtained from PCA dimensionality reduction analysis.

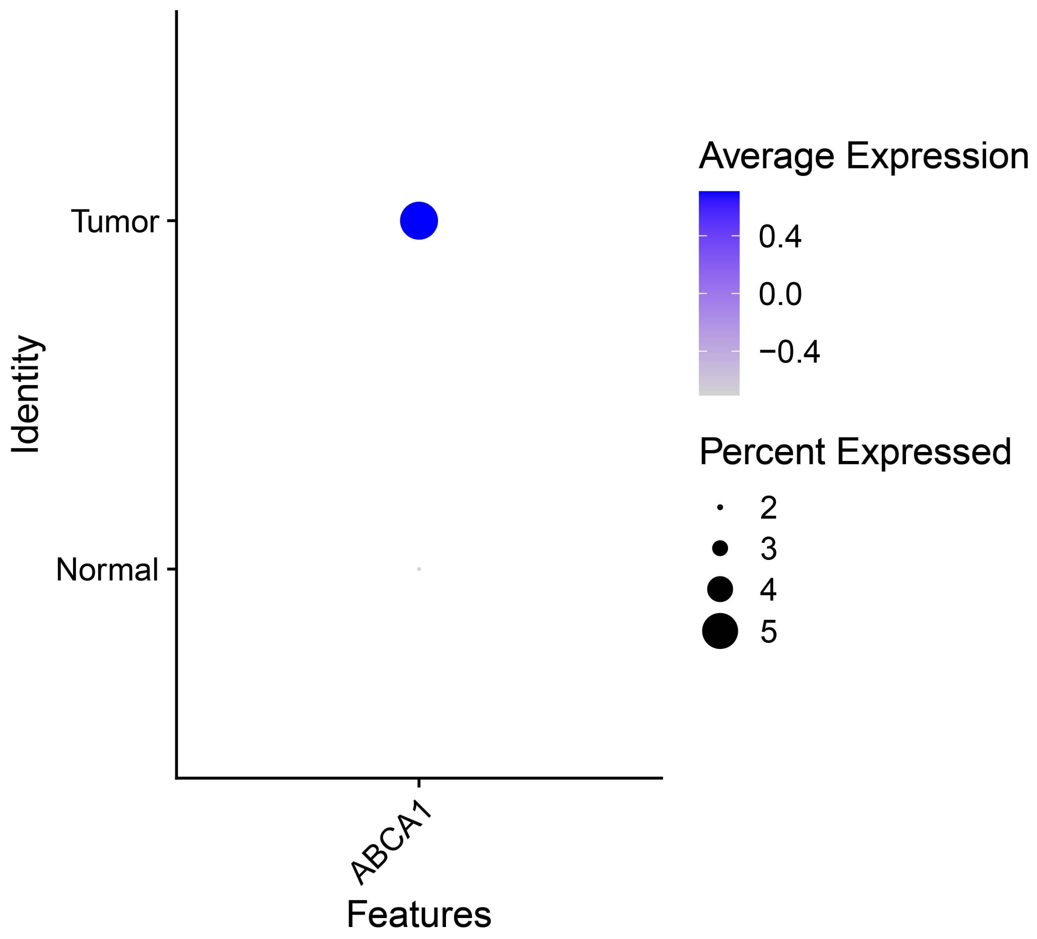

Supplementary Fig. S3 ABCA1 expression in normal and tumour-derived cell subpopulations.
